# Exotic Parasite Threats to Australia’s Biosecurity—Trade, Health, and Conservation

**DOI:** 10.3390/tropicalmed3030076

**Published:** 2018-07-18

**Authors:** R. C. Andrew Thompson

**Affiliations:** School of Veterinary and Life Sciences, Murdoch University, Murdoch WA 6150, Australia; a.thompson@murdoch.edu.au

**Keywords:** Australia, biosecurity, parasites, zoonoses, wildlife

## Abstract

Parasites have threatened Australia’s biosecurity since the early days of European settlement. Tick fever in cattle and liver fluke, along with their invertebrate hosts, and hydatid disease head the list of parasites that are still impacting livestock industries. In addition, there are many parasites that have been introduced that are of significance to public health as well as the conservation of native wildlife. As a consequence of these early arrivals, Australia has become much more aware of its vulnerability should parasites such as *Trichinella* and *Trypanosoma evansi* become established in Australia. However, recent discoveries concerning *Leishmania* and other trypanosomes have demonstrated that Australia must not become complacent and reliant on dogma when considering the potential emergence of new threats to its biosecurity. In this short review, the major parasite threats to Australia’s biosecurity are summarised, some misconceptions are emphasised, and attention is given to the importance of challenging dogma in the face of a dearth of information about Australian native fauna.

## 1. Introduction

Parasites have threatened Australia’s biosecurity since the early days of European settlement. Australia still suffers from some mistakes of the past when parasites were introduced during early settlement [1]. Tick fever in cattle and fascioliasis in livestock, along with their invertebrate hosts, and hydatid disease head the list of exotic parasitic diseases that are still impacting livestock industries. All three diseases were introduced as a consequence of agricultural activities. In addition, there are many introduced parasites that are of significance to public health as well as the conservation of native wildlife that were not endemic to Australia before early settlement.

These early introductions fuelled an awareness of Australia’s vulnerability to exotic pests and parasites, particularly those of economic significance such as *Chrysomya bezziana* (screwworm), *Trypanosoma evansi* (surra), and *Trichinella spiralis* (trichinellosis) [1]. From a public health perspective, only *Plasmodium* seems to be a priority. Australia was declared malaria free in 1981 [2], yet Australia has a vector, *Anopheles farauti* [3]. Parasites such as *Taenia solium*, *Clonorchis sinensis*, and *Trypanosoma cruzi* are unlikely to become established in Australia, yet patients with diseases caused by these parasites migrate to Australia and there is a need for heightened awareness among clinicians about these often chronic infections [4,5].

Furthermore, recent discoveries concerning *Leishmania* and other trypanosomes (see below) have demonstrated that Australia must not become complacent and reliant on dogma when considering the potential emergence of new threats to its biosecurity.

In this review, I want to highlight the major parasite threats to Australia’s biosecurity, some misconceptions, and the importance of challenging dogma in the face of a dearth of information about Australian native fauna.

## 2. Hydatid Disease—*Echinococcus*

*Echinococcus granulosus* is the causative agent of hydatid disease (or, using more recent terminology, cystic echinococcosis) in humans and livestock. It serves as perhaps the best example of a parasite that was brought into Australia in animals that were exotic to the country (principally sheep) [6] but which were essential to the development of livestock agriculture in Australia. Much has been written about the history of hydatid disease in Australia and its control [6]. Unlike the mainland, the island state of Tasmania has been able to effectively control transmission of *E. granulosus* in a dog-sheep cycle [7,8]. Although no new human cases have been reported in Tasmania, complete elimination of the parasite has proved elusive, with recent localised reports in cattle and dogs suggesting transmission may still be occurring, albeit at a low level [9]. However, this is not the case on the mainland of Australia. Tasmania had the advantage of more easily focussing a public health campaign on the dog-sheep cycle and the anthropogenic activities that sustained it, since there was no opportunity of spillover of *Echinococcus* infection to native wildlife, given the absence of the dingo [10]. On the mainland, spillover from the sheep-dog cycle has resulted in a novel cycle of transmission involving small macropods and dingoes [10]. This means that although the incidence of cystic echinococcosis has declined markedly in humans and sheep, the parasite is still maintained in a wild animal cycle and thus complete eradication of this important zoonosis will never be achieved. What was unexpected, however, has been data demonstrating the clinical impact of hydatid disease on native wildlife. Barnes and colleagues [11] investigated the growth rate and clinical presentations of hydatid cysts in macropod marsupials and demonstrated that the cysts have a major impact on respiratory capacity. Infected animals are thus potentially more susceptible to predation by wild dogs, a similar situation to the predator-prey cycle involving moose and large cervids that maintains *E. canadensis* in North America [12]. 

*Echinococcus multilocularis* is not present in Australia nor in Southeast Asia and is largely confined to the northern hemisphere [8]. This could be luck or due to the insusceptibility of native wildlife, because infected dogs are likely to have entered Australia. However, a case report of alveolar echinococcosis in a captive red-necked wallaby (*Macropus rufogriseus*) in Germany [13] demonstrates that Australia’s native marsupials are likely to be susceptible to infection with the metacestode stage of *E. multilocularis*. Consequently, should an infected definitive host enter Australia, most likely a domestic dog, infection could conceivably be transmitted to free-ranging marsupials. Although quarantine restrictions should prevent an infected dog entering Australia, this depends on adequate screening, compliance with Australian quarantine regulations, and use of the most appropriate cestocidal agent. If an infected dog were to enter Australia, the post-arrival period of only 10 days in quarantine from endemic countries such as Switzerland is much less than the pre-patent period [14]. Australian authorities should guard against complacency with a parasite such as *E. multilocularis*. Within recent years, we have seen an increasing number of dogs arriving in Australia from Europe with pathogenic *Leishmania* spp. (see below) and perhaps extra vigilance may be required in post-arrival parasite checks for *E. multilocularis*.

## 3. Economic Threats

From an economic perspective, *Trypanosoma evansi* and *Trichinella spiralis* have always been considered major parasite threats to Australia’s biosecurity [1].

Infection with *T. evansi* can lead to a disease called surra. While currently absent from Australia and Papua New Guinea, it is considered an exotic disease of concern for Australia [15,16,17]. The parasite has a low host specificity, and the clinical consequences of infection can be severe in a range of domestic and some wild animal species, particularly horses, camels, cattle, buffalo, cats, and dogs. In contrast, infection with *T. evansi* leads to mild or chronic disease in sheep and pigs but is often asymptomatic. Experimental evidence suggests infection with *T. evansi* is likely to lead to death in Australian macropod marsupials. *Trypanosoma evansi* has always been considered a major biosecurity risk to Australia because it is transmitted mechanically by tabanid biting flies and is endemic in a number of Southeast Asian regions in close proximity to the north of Australia [17,18]. Since pigs are highly susceptible and rarely suffer overt clinical signs, they can act as ‘silent’ reservoir hosts. This is a major concern given the large numbers of free-ranging feral pigs, particularly in northern Australia. Therefore, both pre- and post-border quarantine surveillance has been undertaken for many years.

Surra was diagnosed in Western Australia in a group of imported camels at Port Hedland in 1907 [19]. These animals were destroyed and there has been no further report of the disease in camels, or any other domestic or wild species of mammal, in Australia since that time. Given the widespread distribution of potential mechanical vectors of *T. evansi* in Australia and the close proximity to endemic areas, it is perhaps surprising that evidence of *T. evansi* has not been detected in Australia. Transmission of infection is related to several factors, including the size (and morphology) of the biting insect (volume of blood potentially transferred from one host to another) and insect density [15]. The latter may be particularly important in areas of northern Australia where the main potential reservoir host, feral pigs, are found. There is perhaps a need to examine vectorial density with that of feral pigs in order to re-evaluate the risk of *T. evansi* establishment in Australia.

*Trichinella* species have been detected worldwide in domestic and wild animals [20]. Economically, *T. spiralis* is the most important species because of its low host specificity and anthropogenic activities that perpetuate a domestic cycle involving pigs. *Trichinella spiralis* has never been reported in Australia, but *T. pseudospiralis* infection has been detected in marsupials and birds in Tasmania [21] and in a human from Tasmania [22]. Australia’s domestic pig population is free from *T. pseudospiralis* and other *Trichinella* species [23]. *Trichinella papuae* has been detected in a mature boar from a Torres Strait island and has been described in pigs in New Guinea [24]. Thus, on current evidence, *Trichinella* does not appear to represent a major biosecurity risk to Australia, although continued surveillance is essential to demonstrate its absence.

## 4. Aboriginal Health

Early settlers brought a variety of parasites with them, including *Ancylostoma*, *Strongyloides*, *Ascaris*, *Trichuris*, *Giardia*, and *Hymenolepis*/*Rodentolepis* [25,26]. Following their introduction to Australia, they all became established in Aboriginal people, who probably represented a naïve host population, and were at particularly high risk of infection as community living was imposed on them. Apart from *Giardia*, these parasites are rarely seen in the general population, whereas they remain endemic in Aboriginal communities, often at high prevalence rates, particularly in children [26]. In all cases, the impact of these parasites is far more severe than in non-Aboriginal people. This is because infections are exacerbated by poor diet, are often chronic, usually involve multiple infections with several parasites, and re-infection is common. Unfortunately, the lack of ongoing surveillance in Aboriginal communities, and thus a lack of current data on the incidence of parasite infections, is a severe impediment to control.

In these situations, control has proved to be very difficult, particularly with parasites transmitted by the faecal-oral route, such as *Giardia* and *Hymenolepis*/*Rodentolepis*, which require improved hygiene and public health measures directed principally at children [26,27,28]. Similarly, although not as widespread, infections with *Ancylostoma* and *Strongyloides* have proved difficult to control once established in communities [25,29]. Control requires not just education but community-wide chemotherapy to reduce prevalence significantly and lead to eradication. Such initiatives have proved very successful for both *Ancylostoma* and *Strongyloides* in the few communities where such mass drug administrations (MDAs) have been undertaken. However, long-term success requires subsequent surveillance of the communities and the implementation of ongoing control strategies when needed. The hookworm trial was conducted over a period of six years and prevalence was reduced from 80% to 2.6% [30], but since then no follow-up prevalence surveys have been reported. The two more recent community MDAs targeting *Strongyloides* were delivered 12 months apart and resulted in a significant sustained reduction in *Strongyloides* seroprevalence over 18 months [31,32]. Recent reports of the occurrence of the zoonotic hookworm *A. ceylanicum* in Australia will further complicate control in Aboriginal communities [33,34]. The parasite has recently been reported in domestic dogs from urban area and Aboriginal communities in northern Australia and is considered an emerging public health risk [35]. Future MDAs aimed at controlling hookworm in communities must therefore take into account the role of dogs in transmission.

With global warming, parasite infections currently endemic in Aboriginal communities may become more common and more widespread within Australia. In addition, the potential for the establishment of introduced infections such as Japanese encephalitis and malaria may also increase [36].

## 5. Trypanosomes

Apart from surra, trypanosomes (*Trypanosoma* and *Leishmania*) have not, until recently, been considered a biosecurity issue for Australia. Although the occurrence of species of *Trypanosoma* in introduced and native wildlife has been documented since the 1950s, their impact as the causative agents of disease was not considered until recently, and attention was principally on documenting host records [16,37]. However, two recent investigations have radically changed our understanding of trypanosomes in Australia, with ramifications internationally. The first was the discovery of a novel species of *Leishmania*, *L. macropodum*, in kangaroos, which is transmitted by a new vector, a biting midge (*Forcipomyia* spp.) [38,39,40]. These discoveries have shattered perceived dogma calling into question the exclusivity of the *Leishmania*-phlebotomine relationship and have been followed by several reports confirming the vectorial potential of biting midges of the genus *Culicoides*, in South America and North Africa [41,42]. The biosecurity implications for Australia are that we have an indigenous *Leishmania*-competent vector in *Forcipomyia* that could transmit pathogenic zoonotic species of *Leishmania*, such as *L. infantum*, that are regularly introduced into Australia in humans and domestic dogs [43,44].

Similarly, *Trypanosoma cruzi* has received little attention in Australia, with the belief that it was restricted to South America where its known triatomid vectors are found. However, recent studies on native Australian species of *Trypanosoma* have not only revealed considerable genetic diversity, with some species closely related to *T. cruzi*, but also that some species are phenotypically similar to *T. cruzi* in their invasive nature at the cellular level, and opportunistic pathogens in some marsupial species contributing to their decline [44,45,46]. Little is known about the vectors of Australian trypanosomes, but the close relationship of some such as *T. noyesi* to *T. cruzi* indicates that their vectors could transmit *T. cruzi*. The vectors of Australian trypanosomes have yet to be identified, although ticks, tabanids, and biting midges may play a role [37,44,47]. Migrants from *T. cruzi*-endemic areas in South America who contracted Chagas’ disease before arriving in Australia could be a source of infection to humans, dogs, or wildlife if local vectors of wildlife species of *Trypanosoma* are capable of transmitting *T. cruzi*.

Ongoing surveillance is required to monitor and address the potential biosecurity concerns of exotic trypanosomes becoming established in Australian mammals [16]. For example, in 1951, forty brush-tailed possums and a single short-beaked echidna were experimentally infected with *T. cruzi*, with the majority of animals dying between 21 and 35 days after inoculation [48]. Given the wide host range of *T. noyesi* in Australia and its genetic relationship with *T. cruzi*, it is possible that the vector of *T. noyesi* could transmit *T. cruzi* from humans (of which there were an estimated 1400–3000 human cases in Australia in 2006 [49,50]) toindigenous Australian mammals. The consequences of infection could lead to disease in naïve wildlife hosts as well as establishing a local reservoir of infection in those animals that do not succumb to infection.

In addition to the pathogenic potential of some Australian trypanosomes to native wildlife, there is increasing evidence that an exotic species of *Trypanosoma*, *T. lewisi*, introduced with rats and their fleas at the time of European settlement, or earlier, could have been responsible for reported declines of native wildlife, in a similar way that its introduction to Christmas Island has recently been found to have been responsible for the extinction of the Maclear’s and bulldog rats [51]. Using ancient DNA methodology, Wyatt et al. [52] demonstrated that native trypanosomes were absent from the indigenous rodents on Christmas Island prior to the introduction of the black rat, and that after 1899 both the Maclear’s and bulldog rat were infected with *T. lewisi* [16]. *Trypanosoma lewisi* was first reported in Australia by Bancroft in 1888 in an introduced *Rattus* sp. [44]. It was subsequently reported in native bush rats and water rats (*Hydromys chrysogaster*) [53]. Its origin within native wildlife is likely to have been from black and brown rats on ships arriving from Europe, or it could have been present before their arrival. Given the pathogenic potential of *T. lewisi* in naïve hosts, as demonstrated on Christmas Island, it has been suggested that *T. lewisi* may have played a role in the decline of native wildlife in Australia [37,44]. Trypanosomes have been demonstrated to vary in virulence when they encounter a new or naïve host species [37,44]. The exotic *T. lewisi* could possibly have played a role in the fauna declines identified in Australia between 1875 and 1925 [54] and since this time a more balanced host/parasite relationship has developed. Investigating the presence of *T. lewisi* in densely populated areas within Australia may assist in answering these questions [37].

Insufficient consideration has been given to the impact of introduced parasites on Australian native wildlife. Although there has been speculation in the past that *Toxoplasma* may have played a role in the decline of some native species of wildlife, recent evidence suggests that *Toxoplasma* may have been in Australia before European settlement and its ubiquity in native wildlife with little evidence of clinical disease in free-ranging wildlife attests to the evolution of a balanced host-parasite relationship [55,56]. It has always been considered that *Toxoplasma* was introduced into Australia in domestic cats and sheep at the time of European settlement [56]. Subsequently, *Toxoplasma* was linked historically to the population declines of native wildlife and has generally been believed to be pathogenic to Australia’s mammalian and avian fauna [54,56]. However, there is little evidence to support these assumptions. The evidence that *Toxoplasma* is a significant cause of disease in native wildlife is largely based on the impact of infection in captive animals [56]. The dogma that *Toxoplasma* is a serious pathogen in Australian wildlife may have overshadowed the potential impact of other parasites such as *T. lewisi*.

## 6. Concluding Comments

Surveillance, pre- and post-border, of both parasites and their vectors, is the key to maintaining Australia’s biosecurity. This has been demonstrated very well with parasites such as *Chrysomya bezziana*. Australia has also recognised which exotic parasites pose threats to Australia’s biosecurity, particularly from an economic perspective. *Trichinella spiralis* and *Trypanosoma evansi* are good examples of parasites that fall into this category, although it is unfortunate that parasites of public health significance are not afforded the same attention. Furthermore, the recent discovery of both a novel species of *Leishmania* and its vector illustrates how little we know about the parasites of Australia’s native fauna. As such, the dangers of local transmission of *Trypanosoma cruzi* in Australia should not be dismissed, given its emergence in the USA and involvement of native vectors. It should not be necessary to wait until a species is in danger of extinction before doing this [57], but such studies should be ongoing and the importance of doing so recognised by appropriate conservation agencies and granting agencies.
